# Mezagitamab in systemic lupus erythematosus: clinical and mechanistic findings of CD38 inhibition in an autoimmune disease

**DOI:** 10.1136/lupus-2023-001112

**Published:** 2024-03-07

**Authors:** Scott R P McDonnell, Van Anh Nguyen, Noah M Walton, Carsten Merkwirth, Feng Hong, Deborah Berg, Elena Tomaselli Muensterman, Richard A Furie

**Affiliations:** 1 Takeda Development Center Americas Inc, Lexington, Massachusetts, USA; 2 Clinical Sciences, Takeda Pharmaceuticals America Inc, Lexington, Massachusetts, USA; 3 Department of Rheumatology, Northwell Health, Great Neck, New York, USA

**Keywords:** systemic lupus erythematosus, interferon type I, antibodies, B-lymphocytes, biological products

## Abstract

**Objective:**

To evaluate safety and mechanism of action of mezagitamab (TAK-079), an anti-CD38 monoclonal antibody, in patients with moderate to severe systemic lupus erythematosus (SLE).

**Methods:**

A phase 1b double-blind, placebo-controlled, multicentre study was conducted in patients with SLE receiving standard background therapy. Eligible patients were adults who met the 2012 SLICC or ACR criteria for diagnosis, had a baseline SLE Disease Activity Index 2000 (SLEDAI-2K) score of ≥6 and were positive for anti-double-stranded DNA antibodies and/or anti-extractable nuclear antigens antibodies. Patients received 45 mg, 90 mg or 135 mg of mezagitamab or placebo every 3 weeks over 12 weeks. Primary endpoints were safety and tolerability. Secondary endpoints included pharmacokinetics and pharmacodynamics. Exploratory assessments included disease activity scales, deep immune profiling and interferon pathway analysis.

**Results:**

22 patients received at least one dose of either mezagitamab or placebo. In patients exposed to mezagitamab (n=17), drug was well tolerated. Adverse event (AEs) were balanced across treatment groups, with no treatment emergent AEs exceeding grade 2. Responder analyses for Cutaneous Lupus Erythematosus Disease Area and Severity Index (CLASI) and SLEDAI-2K did not reveal any observable differences across treatment groups. However, there was a trend for more profound skin responses among patients with higher CLASI scores (>10) at baseline. Pharmacodynamic analysis showed median CD38 receptor occupancy up to 88.4% on CD38+ natural killer cells with concurrent depletion of these cells up to 90% in the 135 mg group. Mean reductions in IgG and autoantibodies were less than 20% in all dose groups. Cytometry by time of flight and type 1 interferon gene analysis revealed unique fingerprints that are indicative of a broad immune landscape shift following CD38 targeting.

**Conclusions:**

Mezagitamab had a favourable safety profile in patients with moderate to severe SLE and elicited a pharmacodynamic effect consistent with CD38+ cell depletion. These findings reveal novel insights into the drug’s mechanism of action and support the continued investigation of mezagitamab in autoimmune diseases.

**WHAT IS ALREADY KNOWN ON THIS TOPIC**

- High expression of CD38 on leukocytes of systemic lupus erythematosus (SLE) patients makes it a potential treatment target for intervention in this disease.

**WHAT THIS STUDY ADDS**

- Mezagitamab, an anti-CD38 antibody, has a favourable safety profile in patients with moderate to severe SLE.
- Mezagitamab induces broad immune landscape changes consistent with CD38+ cell depletion and reduction in type 1 interferon signalling.

**HOW THIS STUDY MIGHT AFFECT RESEARCH, PRACTICE OR POLICY**

- Mezagitamab may have utility in various autoimmune diseases driven by CD38-expressing cells.

## Introduction

Systemic lupus erythematosus (SLE) is a heterogeneous autoimmune disease characterised by dysregulation of cells of T and B-cell lineage as well as other components of the innate immune system.^1 2^ A hallmark of the disease is the production of pathogenic autoantibodies to double-stranded DNA (dsDNA) and/or extractable nuclear antigens (ENA), phospholipids, blood cells and other antigens.^3^ Inflammation leading to tissue damage in SLE is incited primarily by these pathogenic autoantibodies through immune complex deposition and direct antibody–target interactions. SLE can affect virtually any organ in the body.

Most therapeutic agents that are used for treatment of SLE have demonstrated limited success in reducing autoantibodies (particularly anti-ENA antibodies) because they do not specifically target antibody-producing cells.^4^ Short-lived plasmablasts and long-lived plasma cells produce a variety of characteristic autoantibodies and are, therefore, critically involved in SLE pathogenesis. Studies have shown an increased number of plasmablasts in the blood of patients with active SLE.^5^

CD38 is a type II glycoprotein that is highly and uniformly expressed on antibody-producing plasmablasts and plasma cells. In an ex vivo study of CD38 expression on various immune cells in peripheral blood mononuclear cells (PBMCs) from patients with SLE, the highest CD38 expression was observed on plasma cells and plasmablasts, followed by natural killer (NK) cells, plasmacytoid dendritic cells (pDCs), a regulatory T cell subpopulation and naïve T cells.^6^ These findings suggest that CD38 is a well-suited target for therapeutic investigation in SLE.^7^ A case report of daratumumab, an anti-CD38 antibody, in SLE demonstrated preliminary evidence of efficacy and proof of mechanism. In two patients with severe, life-threatening manifestations of SLE, profound clinical responses were accompanied by substantial depletions of autoantibodies, reduction in plasmablasts and decrease in type I interferon (IFN) activity.^8^ Furthermore, the clinical benefits of daratumumab have also been reported in other autoantibody-driven diseases, such as primary Sjorgen disease, anti-neutrophil cytoplasmic antibody-associated vasculitis and immune thrombocytopenia, highlighting the importance of CD38 in autoimmunity.^9^

Mezagitamab (also known as TAK-079) is an investigational fully human immunoglobulin G1 (IgG1) monoclonal antibody (mAb) that binds with high affinity to CD38.^10^ The available non-clinical, first-in-human data and preliminary clinical data in patients with multiple myeloma demonstrated an acceptable safety profile and encouraging pharmacodynamic (PD) effects in reducing target cells expressing CD38.^11–13^ These observations supported investigation of mezagitamab in SLE, a disease characterised by high prevalence of cells with dysregulated CD38 expression. Presented here are the results of the phase 1b trial (NCT03724916) investigating the safety, pharmacokinetics (PK) and PD of mezagitamab in patients with moderate to severe SLE.

## Methods

### Study design

This phase 1b double-blind, placebo-controlled, multicentre study evaluated the safety, PK and PD of mezagitamab across three sequentially enrolling cohorts in a study population receiving standard principal investigator-directed background therapy for persistent moderate to severe SLE (online supplemental figure 1). Each cohort aimed to enrol eight patients in a 3:1 randomisation scheme (mezagitamab: placebo). Patients were assigned to either mezagitamab or placebo administered as a subcutaneous injection every 3 weeks for 12 weeks (total of four doses). Treatment compliance was calculated as (actual number of doses taken)/(planned number of doses)×100. Patients were assessed every 4 weeks for 12 weeks during the safety follow-up period.

10.1136/lupus-2023-001112.supp1 Supplementary data

**Figure 1:**
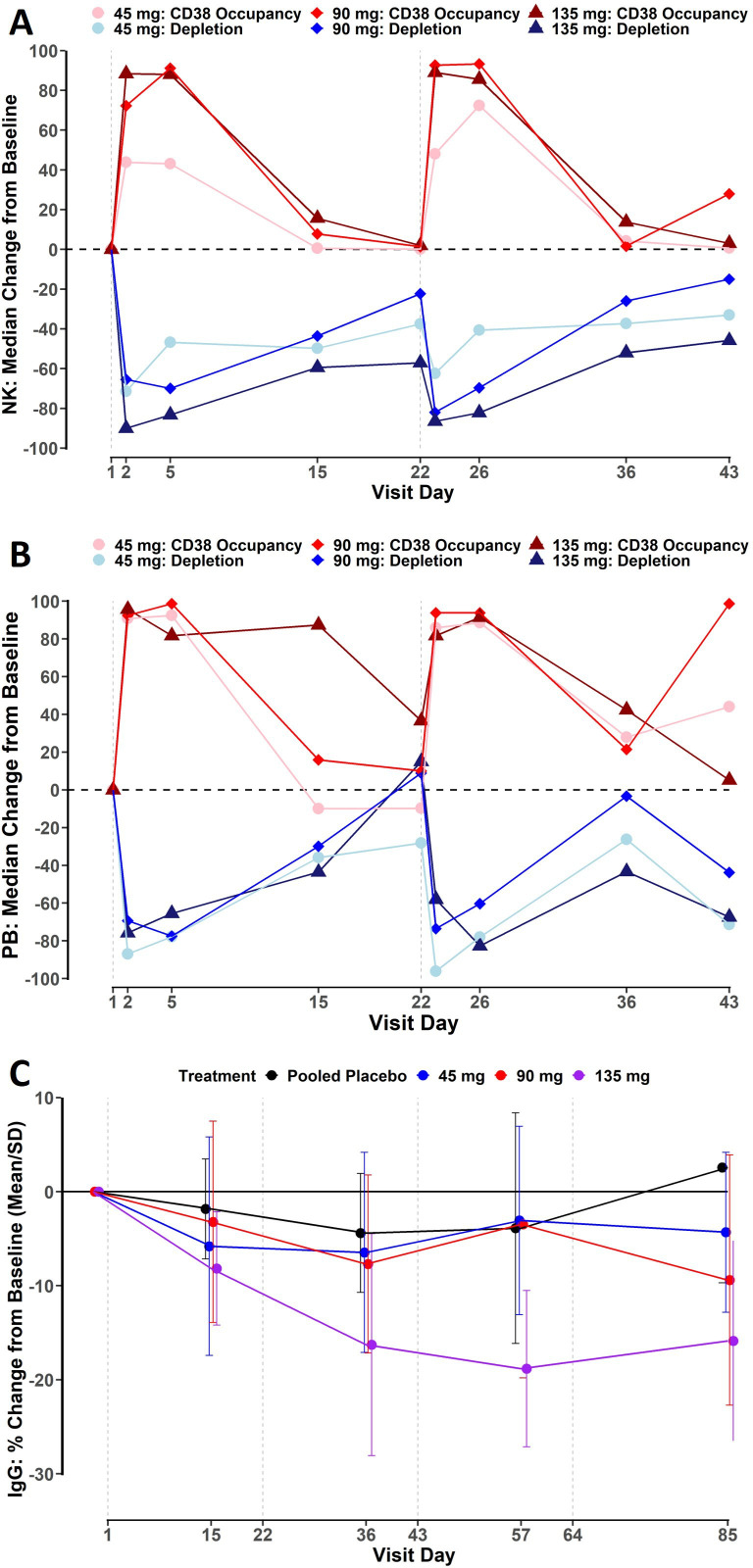
Pharmacodynamics of mezagitamab in patients with SLE. Receptor occupancy and population change in NK cells (A) and plasmablasts (B) isolated from patient blood. Data expressed as median % change from pretreatment baseline. (C) Dose-dependent, longitudinal alterations in serum IgG levels, expressed as mean % change from pretreatment baseline±SD. SLE, systemic lupus erythematosus.

Eligible patients needed to meet either the 2012 Systemic Lupus International Collaborating Clinics classification criteria or the American College of Rheumatology classification criteria for SLE. Additional key inclusion criteria included having SLE Disease Activity Index 2000 (SLEDAI-2K) score ≥6 and being positive for anti-dsDNA antibodies and/or anti-ENA antibodies. Patients were required to receive stable background SLE therapy for ≥12 weeks (with stable dosing for ≥8 weeks) prior to screening and throughout the study. Allowed SLE concomitant medications were immunosuppressants (azathioprine, mycophenolate mofetil and methotrexate), antimalarials and corticosteroids. Key exclusion criteria were a concurrent diagnosis of drug-induced SLE or any concomitant rheumatologic or autoimmune disease that would confound efficacy assessments, active neuropsychiatric SLE or active lupus nephritis documented by an acute flare within 3 months of screening. Additional exclusion criteria were positive hepatitis B surface antigen or hepatitis C antibody or HIV antibody/antigen; an opportunistic infection or an acute or chronic infection requiring hospitalisation within ≤12 weeks and ≤30 days of screening, respectively.

This study was conducted with the highest respect for the individual patients, in accordance with the study protocol, ethical principles of the Declaration of Helsinki, the International Council for Harmonisation of Technical Requirements for Pharmaceuticals for Human Use Harmonised Tripartite Guideline for Good Clinical Practice and all applicable local regulations. The investigator explained the study, including its objectives and potential risks and benefits, to patients using the informed consent form approved by the institutional review boards or independent ethics committees (Advarra Institutional Review Board (Pro00029715), Western Institutional Review Board (2019P000259), Oklahoma Medical Research Foundation (OMRF) (19-10), UCSD Human Research Protections (Project # 190227), Partners Human Research Committee (Protocol # 2019P000964)). Each patient signed and dated the informed consent form before any protocol-specific screening procedures.

Primary endpoints were safety and tolerability, including the incidence, type and grade of adverse events (AEs) as well as the percentage of patients with ≥1 AE, leading to study treatment discontinuation. Secondary endpoints included PK and PD. Exploratory endpoints assessed the effects of repeated administration of mezagitamab on SLE disease activity using clinical rating scales such as SLEDAI-2K and Cutaneous Lupus Erythematosus Disease Area and Severity Index (CLASI) and biomarkers.

The study was not statistically powered for any efficacy hypothesis testing. The sample size for each study cohort was deemed sufficient to fulfil the primary and secondary study objectives in each cohort.

### Receptor occupancy assay

The CD38 receptor occupancy flow cytometry assay was developed to evaluate changes in T cells, B cells, NK cells, monocytes, granulocytes, plasmablasts and plasma cells in whole blood. Additionally, CD38 expression and mezagitamab receptor occupancy were evaluated on the respective cell types by comparing CD38 fluorescence signal for two independent flow cytometry samples containing either labelled mezagitamab (for quantification of ‘free’ CD38 receptor) or labelled TSF-19 (non-competitive CD38 antibody for quantification of ‘total’ CD38 receptor).

### PK, IgG and autoantibody quantification

Mezagitamab serum concentrations were quantified through a clinically validated electrochemiluminescence immunoassay with a lower limit of quantification (LLOQ) of 5 ng/mL. IgG measurements were obtained from Roche Cobas 8000 analysers at a central laboratory. SLE-associated autoantibodies were quantified via Thermo Scientific Phadia 250 Analysers at a central laboratory using clinically validated enzyme-linked immunoassay methodology. Autoantibodies assessed included the following (with positivity defined based on cut-off values): anti-dsDNA (>15 IU/mL); anti-SmD^p^, ribonucleoprotein-70, Sjogrens SS-A, Sjogrens SS-B, beta-2 glycoprotein IgG, beta-2 glycoprotein IgM (>10 IU/mL); cardiolipin antibody IgG and cardiolipin antibody IgM (>40 IU/mL); and cardiolipin antibody IgA (>20 IU/mL).

### Immune profiling

Cytometry by time of flight (CyTOF) analysis was conducted at CellCarta (Fremont, California). Samples were run in batches of approximately 10. Each sample was thawed and washed, stained with a dye indicating cell viability, then hybridised with the defined antibody panel. Following wash, samples were fixed and incubated with a DNA intercalating agent for 3–7 days at 4°C. Fixative was then removed, and samples were suspended in water for CyTOF analysis. 100 000–250 000 events were analysed per sample. In addition to viability assessments, the expression of 39 cellular markers was analysed via metal ion-coupled antibodies. This antibody panel included TSF-19 as a non-competitive antibody against CD38. Samples were obtained at baseline (during screening, up to 28 days prior to mezagitamab initiation) and at days 15, 36, 57 and 85. Data were collected as flow cytometry standard files and assessed as randomly selected analysis of 100 000 cells per patient sample.

Cluster identification and analysis was done using the R package CyTofWorkflow.^14^ FlowSOM^15^ and ConsensusClusterPlus^16^ were used to identify distinct cell populations based on their expression profiles, and results were visualised using t-distributed stochastic neighbour embedding (TSNE) plots.^17^ FlowSOM employed a self-organising map algorithm to cluster cells into metaclusters, and ConsensusClusterPlus employed a consensus clustering approach to identify stable clusters. Additional gated analysis was conducted and expressed as median CD38 expression and percentage of parent population (±SE of mean, where applicable). Statistical analyses and figure generation were conducted in Microsoft Excel, GraphPad Prism V.9, and R. Flow cytometry panels were generated using FlowJo 10.

IFN gene signature expression was evaluated using Nanostring’s nCounter Autoimmune Profiling Panel validated and run at Q2 Genomics (Durham, North Carolina). Whole blood samples collected in PAXgene tube were used for RNA isolation and analysis. Analysis was conducted on nSolver Advanced Analysis Visualizations (NanoString).

## Results

### Patient demographics

Twenty-three out of the planned 24 patients were enrolled and randomised across 13 study sites in the USA. The study was conducted between 26 November 2018 and 4 November 2021; however, enrolment was closed early due to recruitment challenges. One randomised patient withdrew from the study before receiving study drug, and the remaining 22 patients received at least one dose of study drug. Key demographic characteristics are summarised in table 1.

**Table 1:** Patient baseline demographics and disease characteristics

	Pooled placebo (n=5)	Mezagitamab 45 mg (n=6)	Mezagitamab 90 mg (n=6)	Mezagitamab 135 mg (n=5)
Age, years (SD)	36.4 (6.6)	51.0 (22.0)	46.7 (6.5)	49.6 (13.5)
Sex, female, n (%)	5 (100)	6 (100)	5 (83.3)	4 (80)
Baseline weight, kg, mean (SD)	87.3 (17.7)	75.0 (18.4)	85.6 (28.4)	64.6 (8.16)
Ethnicity, n (%)				
Hispanic or Latino	1 (20)	2 (33.3)	0	1 (20)
Non-Hispanic and Latino	4 (80)	4 (66.7)	6 (100)	4 (80)
Race, n (%)*				
American Indian	0	0	0	1 (20)
Asian	0	0	0	1 (20)
Black or African American	2 (40)	4 (66.7)	1 (16.7)	2 (40)
White	3 (60)	2 (33.3)	4 (66.7)	1 (20)
Multiracial	0	0	1 (16.7)	0
SLEDAI-2K at baseline, mean (SD)	8.4 (1.67)	9.7 (4.27)	9.7 (3.67)	8.8 (1.79)
CLASI at baseline, mean (SD)	4.8 (4.76)	7.2 (5.81)	5.2 (4.62)	11.8 (11.37)
SLE background treatment				
Antimalarials, n (%)	4 (80.0)	5 (83.3)	6 (100)	4 (80.0)
Hydroxychloroquine	3 (60.0)	3 (50.0)	4 (66.7)	3 (60.0)
Hydroxychloroquine sulphate	1 (20.0)	2 (33.3)	2 (33.3)	1 (20.0)
Corticosteroids, n (%)(mean dose (mg))	3 (60.0) (8.3)	2 (33.3) (10)	2 (33.3) (7)	3 (60.0) (4.7)
Prednisone	3 (60.0)	2 (33.3)	1 (16.7)	3 (60.0)
Methylprednisolone	0	0	1 (16.7)	0
Mycophenolate, n (%)	1 (20.0)	2 (33)	1 (16.7)	3 (60.0)
Mycophenolate mofetil	1 (20.0)	1 (16.7)	0	2 (40.0)
Mycophenolate sodium	0	0	1 (16.7)	0
Mycophenolic acid	0	1 (16.7)	0	1 (20.0)
Methotrexate, n (%)	1 (20.0)	2 (33.3)	0	0

*Subject may have more than one race. Those subjects selecting multiple races are counted only under ‘multiracial’.

CLASI, Cutaneous Lupus Erythematosus Disease Area and Severity Index; SLE, systemic lupus erythematosus; SLEDAI-2K, SLE Disease Activity Index 2000.

Demographic variables were generally similar across treatment groups, with the exception of baseline weight and age. Placebo-treated patients were on average approximately 13 years younger (36.4±6.6 years) compared with mezagitamab-treated patients (pooled mezagitamab group: 49.1±14.6 years). The average weight of patients in the mezagitamab 135 mg group was less than in the other treatment arms (placebo, mezagitamab 45 mg or 90 mg). Most patients were women, and there was a similar distribution of African American and Caucasian study participants. All patients were taking at least one background SLE medication, with the most common being hydroxychloroquine (n=13), prednisone (n=9) and mycophenolate mofetil (n=4). There were no changes to background medications during the treatment period. Among those patients receiving corticosteroids, slightly higher mean daily doses were observed in the placebo (8.3 mg) and mezagitamab 45 mg (10.0 mg) groups compared with the mezagitamab 90 mg (7.0 mg) and 135 mg (4.7 mg) groups during the treatment period of the study. Overall, use of background medications was balanced across treatment groups and consistent with the standard of care practices for patients with SLE.

### Safety

In total, 17 patients were exposed to mezagitamab, and 5 patients were exposed to placebo. Mean (range) compliance was 80.0% (50.0%–100.0%) in the pooled placebo group and 79.2% (50.0%–100.0%), 62.5% (25.0%–100.0%) and 70.0% (50.0%–100.0%) in the 45 mg, 90 mg and 135 mg mezagitamab arms, respectively (table 2).

**Table 2:** Overview of compliance and TEAEs by treatment group

	Pooled placebo (N=5)	Mezagitamab 45 mg (N=6)	Mezagitamab 90 mg (N=6)	Mezagitamab 135 mg (N=5)
Treatment compliance (%)*				
Mean (SD)	80.0 (20.9)	79.2 (18.8)	62.5 (34.5)	70.0 (20.9)
Median	75.0	75.0	62.5	75.0
Min, Max	50, 100	50, 100	25, 100	50, 100
**TEAE**				
Any TEAE (% of patients)	3 (60.0)	4 (66.7)	6 (100.0)	2 (40.0)
Grade 3 or higher TEAE	0	0	0	0
Drug-related TEAE (% of patients)	1 (20.0)	1 (16.7)	1 (16.7)	1 (20.0)
Drug-related grade 3 or higher TEAE	0	0	0	0
Treatment-emergent SAEs (% of patients)	0	1 (16.7)	0	1 (20.0)
Drug-related treatment-emergent SAEs	0	0	0	0
TEAEs resulting in study drug dose modification† (% of patients)	0	1 (16.7)	0	1 (20.0)
TEAEs resulting in study drug discontinuation (% of patients)	0	0	0	1 (20.0)
Deaths	0	0	0	0

*Treatment compliance (%) was calculated as (actual number of doses taken)/(planned number of doses) × 100.

†Dose modification includes dose interrupted and drug withdrawn.

SAE, serious adverse event; TEAE, treatment-emergent adverse event.

Many patients did not receive all four doses of study drug (60% in the pooled placebo group and 70.6% in the pooled mezagitamab group) due to a number of factors, including the COVID-19 pandemic and protocol pre-specified dose holds for safety.

Overall, mezagitamab was well tolerated, with no safety concerns identified during the study. There were no substantial imbalances in treatment emergent AEs (TEAEs) across treatment groups (table 2). One patient in each treatment group experienced at least one drug-related TEAE, as determined by the investigator; these included urinary tract infection, nausea, fever and rash. All TEAEs observed in the study had a maximum intensity of grade 1 or grade 2 based on Common Terminology Criteria for Adverse Events. There were two treatment-emergent serious AEs with mezagatimab: palpitations (1 patient in 45 mg group) and dyspnoea (1 patient in 135 mg group; led to study drug withdrawal); neither were considered related to mezagitamab. No cytokine release syndrome events or injection site reaction TEAEs were reported. There was one report of hypersensitivity reaction in the mezagitamab 135 mg group, which consisted of fever with onset on the first administration of study drug and resolved within 1 day. No remarkable findings for laboratory tests, ECGs, vital signs or physical examinations were reported that were related to mezagitamab treatment. There were no trends for increased safety-related dose holds or lower treatment compliance among mezagitamab-treated groups, as only one TEAE (dyspnoea) led to drug discontinuation in the 135 mg group (described above).

### Efficacy

An exploratory objective of this study was to assess the effects of mezagitamab on disease activity using SLE disease activity instruments. At baseline, the mean SLEDAI-2K total scores were similar in the placebo (8.4), mezagitamab 45 mg (9.7), mezagitamab 90 mg (9.7) and mezagitamab 135 mg (8.8) groups (table 1). Mild to moderate improvement in SLEDAI-2K total scores was observed with mezagitamab treatment through the end of treatment without observable differences between treatment groups (online supplemental table 1). At the end of treatment on day 85, the changes from baseline in total scores (LS mean) were comparable in the placebo group (−5.2) and the mezagitamab treatment groups (mezagitamab 45 mg: −3.1; mezagitamab 90 mg: −2.5; mezagitamab 135 mg: −4.2). The numbers of responders, defined as patients whose disease activity score decreased from baseline by at least four points, were similar across treatment groups at day 85. No trends were observed in individual SLEDAI-2K total scores.

At baseline, the mean CLASI total activity scores were lower in the placebo (4.8), mezagitamab 45 mg (7.2) and mezagitamab 90 mg (5.2) groups compared with the mezagitamab 135 mg (11.8) group. There was moderate improvement from baseline to the end of treatment in the CLASI total activity score but with no observable differences between the treatment groups (online supplemental table 1). At the end of treatment on day 85, the change from baseline in total activity score (LS mean) was similar in the placebo group (−3.7) compared with the mezagitamab treatment groups (mezagitamab 45 mg: −4.3; mezagitamab 90 mg: −3.9; mezagitamab 135 mg: −3.6). The numbers of responders, defined as patients whose score decreased from baseline either by at least 4 points or by at least 20%, were also similar across treatment groups at this time point. However, there was a trend for clinically meaningful improvement for patients with more severe skin disease at baseline: all patients with a baseline CLASI score of >10 met responder criteria at end of treatment (online supplemental figure 2). Of these, only one patient was a responder in the placebo group while there were five responders in mezagitamab-treated groups.

**Figure 2:**
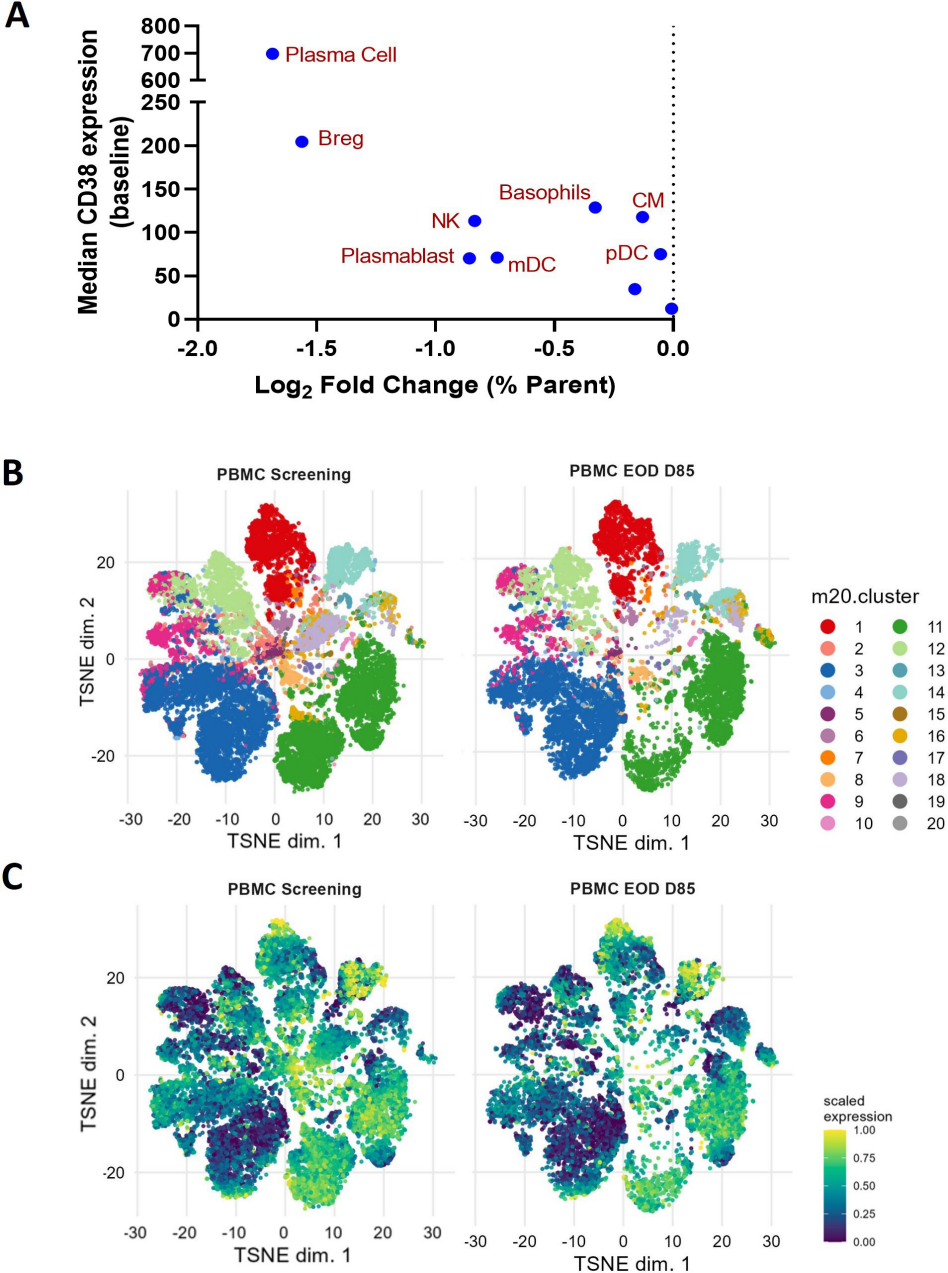
Mezagitamab depleted populations in a roughly CD38-dependent fashion. (A) Depletion of selected CD38 positive cell populations (labelled) plotted against baseline CD38 expression. (B) 20-cluster TSNE cluster mapping identified following CyTOF analysis reveals discrete populations (described in online supplemental figure 5) that are maintained over the course of study. (C) CD38 heatmap expression throughout study. CM, classical monocytes; mDC, myeloid dendritic cells; NK, natural killer; pDCs, plasmacytoid dendritic cells; TSNE, t-distributed stochastic neighbour embedding.

### Pharmacokinetics and pharmacodynamics

Serum concentrations of mezagitamab were detectable in all patients at all dose levels, but below the LLOQ postdose in several patients, particularly in the 45 mg dose group. Peak exposure was greater than dose proportional over the dose range tested (online supplemental figure 3). After the first administration, a threefold increase in dose resulted in an approximately 100-fold increase in mean C_max_ from 57.5 ng/mL to 6130 ng/mL. Most patients reached maximum drug concentrations at 108 hours after the first and second doses of mezagitamab.

The NK cell population is an abundant CD38-expressing cell population in peripheral blood^18^ and, therefore, can be used as a surrogate marker for CD38 engagement on target cells. Mezagitamab engaged the CD38 target on CD38+ NK cells in a dose-dependent manner, with median receptor occupancy increasing from 43.8% to 88.4% in the study dose range of 45–135 mg 1 day after the first dose. Corresponding reductions in NK cells were approximately similar for all mezagitamab dose groups, with −71.5%, −65.5% and −90.0% median changes from baseline observed for 45 mg, 90 mg and 135 mg, respectively (figure 1A). Placebo-treated patients showed no reductions from baseline in CD38+ NK cells throughout the study.

Reductions in absolute plasmablast counts were similar across mezagitamab dose groups. The maximum effects were observed 1 day after the first dose with −87.0%, −69.4% and −75.8% median changes from baseline for 45 mg, 90 mg and 135 mg, respectively (figure 1B). Plasmablast counts returned to baseline values before the second dose administration.

### Downstream pharmacology of targeting CD38

Since plasma cells reside predominantly in tissues (particularly in bone marrow),^19^ potential effects of mezagitamab were assessed indirectly by using serum IgG as a surrogate biomarker. Multiple administrations of either 45 mg or 90 mg doses of mezagitamab resulted in modest reductions in IgG of less than 10% mean decrease from baseline at any given time point and did not show substantial differences compared with the placebo group, which had approximately 5% maximum mean decrease from baseline during the dosing period (figure 1C). In the 135 mg dose group, mezagitamab treatment resulted in 18.8% maximum mean decrease from baseline in IgG. Reductions in immunoglobulins generally did not return to baseline levels by day 85, which was the last time point of the study.

In addition to changes in serum immunoglobulin concentrations, the effects of mezagitamab were assessed for changes in serum concentrations of autoantibodies in patients positive for a given autoantibody at baseline. There were six autoantibodies for which patients were positive: anti-dsDNA, anti-SmD^p^, beta-2 glycoprotein IgM, ribonucleoprotein-70, SS-A and SS-B. Many patients were positive for anti-dsDNA (9 of 22 patients) or SS-A (14 of total 22 patients). Changes in autoantibody concentrations did not appear to be dose dependent and generally did not show strong concordance with changes in total immunoglobulins or clinical response (online supplemental figure 4). Maximum reductions in autoantibody concentrations were approximately 20% mean decrease from baseline for all evaluated autoantibodies (data not shown).

### Cytometry by time of flight

To gain further insight into the broad immune landscape changes induced by mezagitamab, CyTOF analysis was performed on PBMCs. These results showed a general trend of CD38 expression being correlated with the extent of cell depletion (figure 2A). Plasma cells, regulatory B cells (Bregs), NK cells and plasmablasts were among the most impacted populations, in alignment with the receptor occupancy data for a subset of these cells. Additionally, the unsorted CyTOF data were subjected to FlowSOM clustering and TSNE visualisation (figure 2B,C, online supplemental figure 5). These data showed changes in cluster size specifically around clusters expressing high levels of CD38. To obtain more information on specific clusters undergoing substantial alteration, cluster number was increased from 20 to 50 (online supplemental figure 6A). This more granular analysis revealed two clusters of CD8+ and CD4+ (32 and 34, online supplemental figure 6B,C) that express granzyme, are CCR7−, and are CD45rAlo/−. Assessing treatment effect on this population revealed an increase in the prevalence of these populations, which appeared to dependent on time on treatment and/or dose (online supplemental figure 6B,C). Taken together, data showed that mezagitamab targets high CD38-expressing cells, resulting in their depletion and overall reduction of the CD38 signal in immune cells.

### IFN gene signature

**Figure 3:**
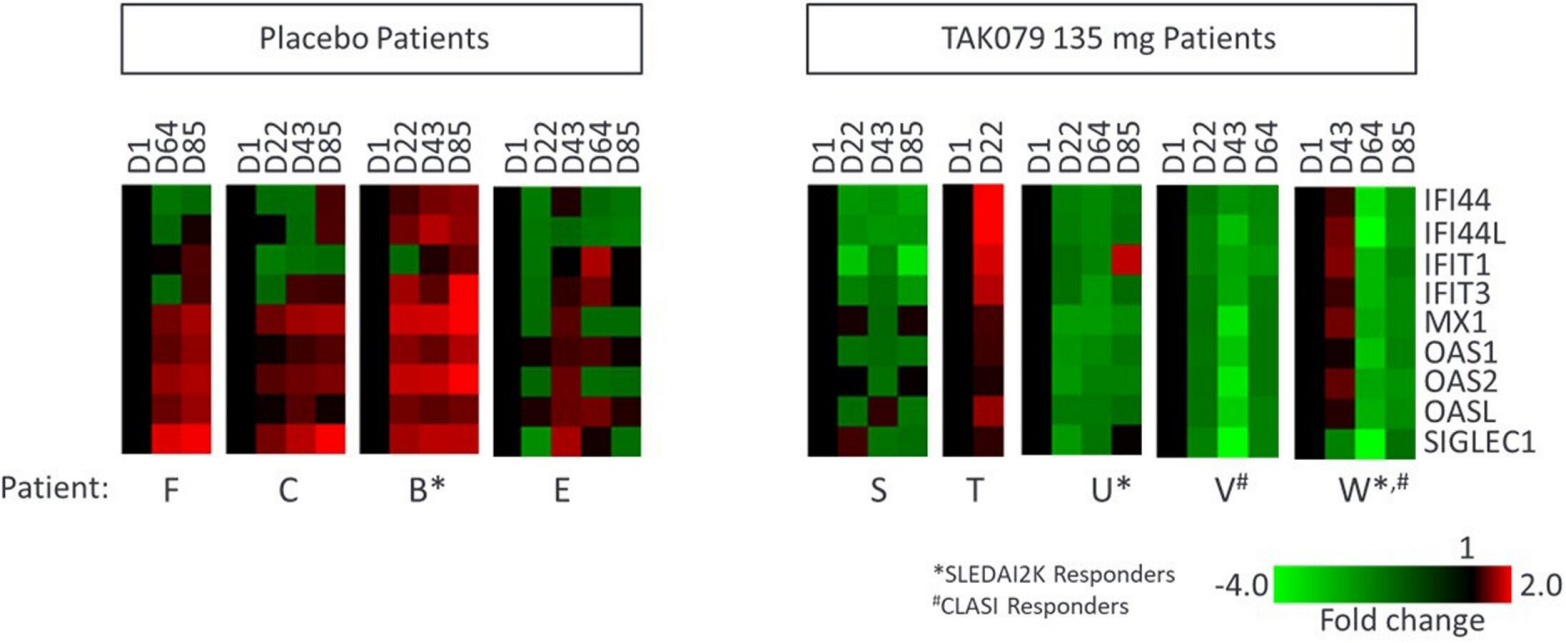
Mezagitamab decreased type 1 interferon responsive genes. A subset of placebo and 135 mg patients had available samples analysed for inflammation-associated RNA gene expression by Nanostring. Data from selected type 1 interferon genes are presented as heatmaps normalised to the baseline expression of each gene for each patient. CLASI, Cutaneous Lupus Erythematosus Disease Area and Severity Index; SLEDAI-2K, SLE Disease Activity Index 2000.

Whole blood RNA samples were collected at discrete timepoints throughout the treatment course and subjected to Nanostring’s autoimmune profiling panel. All available data are presented in figure 3. Viable RNA samples for analysis were available for only a subset of patients in placebo and 135 mg groups and only at indicated time points. Based on the limited data available from placebo (n=4) and 135 mg (n=5), genes associated with a type 1 IFN response appeared to be downregulated in patients in the 135 mg cohort but not in placebo patients (figure 3). This response was most pronounced in the patients with the largest changes in CLASI scoring from baseline.

## Discussion

Mezagitamab was well tolerated, with no safety concerns and no observable differences in safety events across treatment groups identified in this study. The mezagitamab safety results were consistent with those observed in the first-in-human study,^12^ which informed the dose selection in the present study. The starting dose of 45 mg for the initial dosing cohort (ie, cohort A) was selected based on the favourable safety profile and PD target effect (ie, a sustained reduction of plasmablasts) observed after 0.6 mg/kg dose was administered to healthy participants. Because levels of CD38-expressing plasmablasts are generally higher in patients with SLE compared with healthy volunteers,^20^ higher doses were selected for subsequent cohorts to achieve desired PD effects.

Target engagement of mezagitamab on CD38 antigen was evaluated via receptor occupancy on CD38+ NK cells, which serves as a surrogate PD marker due to the abundance in peripheral blood and high expression of the receptor.^18^ Target engagement by mezagitamab on CD38+ NK cells was dose dependent, but not saturated at the doses tested. Return to baseline for receptor occupancy on CD38+ NK cells paralleled partial recovery of this cell population by the time of the next dose administration. Given the importance of these effector cells in driving antibody-dependent cellular cytotoxicity, their recovery is essential for mezagitamab’s cytotoxicity against antibody-producing cells.

The therapeutic hypothesis for investigating mezagitamab in SLE was based on targeting CD38-expressing cells, such as plasma cells and plasmablasts, thus reducing the production of all types of pathogenic autoantibodies that could be present in patients. Reductions in autoantibodies for which patients were positive at baseline did not appear to be correlated with efficacy measures as assessed by disease activity instruments, such as CLASI and SLEDAI-2K—a finding consistent with published literature.^21 22^ Lack of concordance could also have been due to insufficient depletion of plasma cells resulting in limited reductions in immunoglobulins. In addition, the small size of the study and heterogeneity of disease manifestations could have contributed. Since each autoantibody may have a distinct pathogenic contribution to disease activity^3^ and a number of patients were positive for more than one autoantibody, a uniform suppression of all autoantibodies may be required to evaluate the relationship between autoantibody reduction and clinical improvement. These observations, coupled with findings from the receptor occupancy assay, suggest that the dosing regimen of mezagitamab in SLE was not optimal to produce maximal pharmacological effects.

Despite the small sample size and the heterogeneity of the study population, an exploratory objective was to assess the effects of mezagitamab on disease activity using conventional SLE disease activity instruments. The study was not powered to formally compare changes in clinical scores across groups. In addition, efficacy analyses were further hampered because of the COVID-19 pandemic, which resulted in a high number of discontinuations and missed doses, further reducing the number of available patients for efficacy assessments within each cohort. Furthermore, as with most SLE studies, patients were on multiple concomitant medications, including oral corticosteroids, which were not tapered during the study, thus contributing to an inflated treatment response. This was a limitation and was evident by the relatively high placebo response observed in this study.

Clinical response (particularly in CLASI score) in the absence of immunoglobulin reductions in some patients warranted investigation into consequences of CD38 inhibition that are unrelated to antibody-producing target cells. In addition to NK cell depletion observed in the receptor occupancy assay, CyTOF analysis identified effects on other immune cell populations, including reductions in Bregs. Changes in these upstream regulatory cell types may have significant impact on effector cells and more broadly on the immune landscape. To this end, unbiased cluster analysis identified (among others) two clusters representing populations with features of effector CD4 and CD8 T cells, which have been implicated in SLE pathophysiology. Both populations appear to increase in response to treatment, although this was most pronounced only at the highest tested dose, highlighting again that an optimised dosing regimen for mezagitamab in SLE could further increase its pharmacological effects.

Assessment of biomarkers associated with cutaneous lupus was undertaken to gain a mechanistic understanding of profound CLASI responses. Available results indicated apparent reduction in whole blood type I IFN gene expression in mezagitamab 135 mg-treated patients over the course of the trial compared with placebo-treated patients in whom reductions were not observed. As skin biopsies were not performed in this trial, evaluation of tissue IFN expression and tissue-resident immune cells implicated in its production (such as pDCs and keratinocytes) could not be conducted. Since mezagitamab did not impact pDC levels in whole blood, additional investigation is needed to elucidate a link between mezagitamab’s mechanism of action, the observed reduction in IFN gene signature and the CLASI responses.

Overall, this phase 1b study demonstrated mezagitamab’s favourable safety profile, expected PD effects and encouraging mechanistic data in patients with moderate to severe SLE. These findings support continued investigation of mezagitamab in autoimmune diseases.

## Data Availability

Data are available upon reasonable request. The datasets, including the redacted study protocol, redacted statistical analysis plan, and individual participants data supporting the results reported in this article, will be made available within three months from initial request, to researchers who provide a methodologically sound proposal. The data will be provided after its de-identification, in compliance with applicable privacy laws, data protection and requirements for consent and anonymisation.

## References

[1] Li H , Boulougoura A , Endo Y , et al . Abnormalities of T cells in systemic lupus erythematosus: new insights in pathogenesis and therapeutic strategies. J Autoimmun 2022;132:S0896-8411(22)00078-6. 10.1016/j.jaut.2022.102870 35872102

[2] Ma K , Du W , Wang X , et al . Multiple functions of B cells in the pathogenesis of systemic lupus erythematosus. Int J Mol Sci 2019;20:6021. 10.3390/ijms20236021 31795353 PMC6929160

[3] Dema B , Charles N . Autoantibodies in SLE: Specificities, isotypes and receptors. Antibodies (Basel) 2016;5:2. 10.3390/antib5010002 31557984 PMC6698872

[4] Liu Z , Zou Y , Davidson A . Plasma cells in systemic lupus erythematosus: the long and short of it all. Eur J Immunol 2011;41:588–91. 10.1002/eji.201041354 21341259

[5] Dörner T , Giesecke C , Lipsky PE . Mechanisms of B cell Autoimmunity in SLE. Arthritis Res Ther 2011;13:243. 10.1186/ar3433 22078750 PMC3308063

[6] Burns M , Ostendorf L , Biesen R , et al . Dysregulated Cd38 expression on peripheral blood immune cell Subsets in SLE. Int J Mol Sci 2021;22:2424. 10.3390/ijms22052424 33670902 PMC7957821

[7] Parodis I , Gatto M , Sjöwall C . B cells in systemic lupus erythematosus: targets of new therapies and surveillance tools. Front Med (Lausanne) 2022;9:952304. 10.3389/fmed.2022.952304 36111105 PMC9468481

[8] Ostendorf L , Burns M , Durek P , et al . Targeting Cd38 with Daratumumab in refractory systemic lupus erythematosus. N Engl J Med 2020;383:1149–55. 10.1056/NEJMoa2023325 32937047

[9] Holzer M-T , Ruffer N , Huber TB , et al . Daratumumab for autoimmune diseases: a systematic review. RMD Open 2023;9:e003604. 10.1136/rmdopen-2023-003604 38101819 PMC10729190

[10] Smithson G , Zalevsky J , Korver W , et al . TAK-079 is a high affinity Monoclonal antibody that effectively mediates Cd38+ cell depletion. J Immunol 2017;198:224. 10.4049/jimmunol.198.Supp.224.20

[11] Korver W , Carsillo M , Yuan J , et al . A reduction in B, T, and natural killer cells expressing Cd38 by TAK-079 inhibits the induction and progression of collagen-induced arthritis in cynomolgus monkeys. J Pharmacol Exp Ther 2019;370:182–96. 10.1124/jpet.119.256602 31085699

[12] Fedyk ER , Zhao L , Koch A , et al . Safety, tolerability, pharmacokinetics and pharmacodynamics of the anti-Cd38 Cytolytic antibody TAK-079 in healthy subjects. Br J Clin Pharmacol 2020;86:1314–25. 10.1111/bcp.14241 32045493 PMC7319013

[13] Krishnan AY , Patel KK , Hari P , et al . Preliminary results from a phase 1B study of TAK-079, an investigational anti-Cd38 Monoclonal antibody (mAb) in patients with Relapsed/ refractory multiple myeloma (RRMM). Blood 2019;134:140. 10.1182/blood-2019-128007

[14] Nowicka M , Krieg C , Crowell HL , et al . Cytof Workflow: differential discovery in high-throughput high-dimensional Cytometry Datasets. F1000Res 2017;6:748. 10.12688/f1000research.11622.3 28663787 PMC5473464

[15] Van Gassen S , Callebaut B , Van Helden MJ , et al . Flowsom: using self-organizing maps for visualization and interpretation of Cytometry data. Cytometry A 2015;87:636–45. 10.1002/cyto.a.22625 25573116

[16] Wilkerson MD , Hayes DN . Consensusclusterplus: a class discovery tool with confidence assessments and item tracking. Bioinformatics 2010;26:1572–3. 10.1093/bioinformatics/btq170 20427518 PMC2881355

[17] Amir ED , Davis KL , Tadmor MD , et al . viSNE enables visualization of high dimensional single-cell data and reveals Phenotypic heterogeneity of leukemia. Nat Biotechnol 2013;31:545–52. 10.1038/nbt.2594 23685480 PMC4076922

[18] Nagler A , Lanier LL , Cwirla S , et al . Comparative studies of human Fcriii-positive and negative natural killer cells. J Immunol 1989;143:3183–91. 2530273

[19] Nutt SL , Hodgkin PD , Tarlinton DM , et al . The generation of antibody-Secreting plasma cells. Nat Rev Immunol 2015;15:160–71. 10.1038/nri3795 25698678

[20] Cole S , Walsh A , Yin X , et al . Integrative analysis reveals Cd38 as a therapeutic target for plasma cell-rich pre-disease and established rheumatoid arthritis and systemic lupus erythematosus. Arthritis Res Ther 2018;20:85. 10.1186/s13075-018-1578-z 29720240 PMC5932888

[21] Marks SD , Tullus K . Autoantibodies in systemic lupus erythematosus. Pediatr Nephrol 2012;27:1855–68. 10.1007/s00467-011-2078-4 22193636

[22] Pisetsky DS . Evolving story of Autoantibodies in systemic lupus erythematosus. J Autoimmun 2020;110:S0896-8411(19)30714-0. 10.1016/j.jaut.2019.102356 PMC8284812 31810857

