# Supplementary material for: Mezagitamab in systemic lupus erythematosus: clinical and mechanistic findings of CD38 inhibition in an autoimmune disease
Source: Lupus Sci Med. 2024 Mar 7;11(1):e001112. doi: 10.1136/lupus-2023-001112 (PMC10921479; doi:10.1136/lupus-2023-001112)
Supplement: Supplementary data [file lupus-2023-001112supp001.pdf]

Supp. Figure 1: Trial design schematic

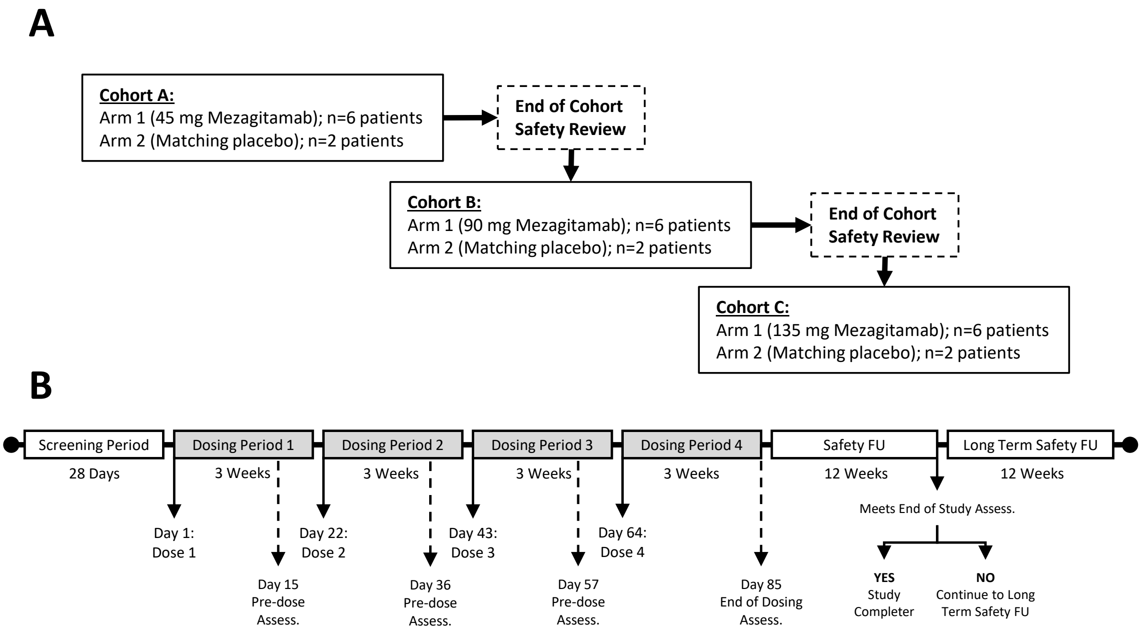

This Phase 1b study compared active mezagitamab with matching placebo in combination with a background SLE standard treatment across 3 sequentially enrolling cohorts in a double-blinded study design, with safety review at the end of each cohort and 3:1 randomization to mezagitamab:placebo (A). The study included a 28-day screening period; 12-week treatment period with single SC dose injection administer every 3 weeks (total of 4 doses); a 12-week safety follow-up (FU) period; and a 12-week long term safety follow-up period (B).

Supp. Table 1: CLASI and SLEDAI-2K efficacy summary

Clinical Score Parameter	Pooled Placebo (N = 5)	TAK-079 45 mg (N = 6)	TAK-079 90 mg (N = 6)	TAK-079 135 mg (N = 5)
**CLASI (Total Activity Score) Baseline; Observed values**				
Mean (SD)	4.8 (4.76)	7.2 (5.81)	5.2 (4.62)	11.8 (11.37)
Median	4.0	6.0	4.5	5.0
Min, Max	1, 13	2, 17	0, 13	3, 27
**CLASI change from baseline at Day 85**				
LS Mean (SEM)	-3.7 (1.56)	-4.3 (1.39)	-3.9 (1.53)	-3.6 (1.61)
Difference in LS Mean (SEM)		-0.6 (2.09)	-0.2 (2.12)	0.1 (2.31)
95% CI of the Difference		-4.9, 3.7	-4.5, 4.1	-4.6, 4.8
**Patients meeting CLASI clinical response criteria**				
n	5	6	4	5
Responders [a]	2 (40.0)	3 (50.0)	2 (50.0)	3 (60.0)
**SLEDAI-2K Baseline; Observed values**				
Mean (SD)	8.4 (1.67)	9.7 (4.27)	9.7 (3.67)	8.8 (1.79)
Median	8.0	8.0	10.0	8.0
Min, Max	6, 10	6, 18	6, 16	8, 12
**SLEDAI-2K change from baseline at Day 85**				
LS Mean (SEM)	-5.2 (1.30)	-3.1 (1.17)	-2.5 (1.29)	-4.2 (1.29)
Difference in LS Mean (SEM)		2.0 (1.76)	2.7 (1.85)	0.9 (1.82)
95% CI of the Difference		(-1.5, 5.6)	(-1.0, 6.4)	(-2.7, 4.6)
**Patients meeting SLEDAI-2K clinical response criteria**				
n	5	6	5	5
Responders [b]	3 (60.0)	3 (50.0)	3 (60.0)	2 (40.0)

CLASI: Cutaneous Lupus Erythematosus Disease Area and Severity Index; SLEDAI-2K: Systemic Lupus Erythematosus Disease Activity Index 2000; SD: standard deviation; LS: least squares; SEM: standard error of the mean; CI: confidence interval.

[a] Responder is defined as a patient whose score decreased from baseline either by at least 4 points or by at least 20%.
[b] Responder is defined as a patient whose score decreased from baseline by at least 4 points.

Supp. Figure 2: CLASI responder analysis

Patient ID	Study Group	Baseline Scores	Baseline Clinical Presentation	EOT Scores	EOT Clinical Presentation	Δ
A	Placebo	13	Verrucous hypertrophic dyspigmentation on nose, red erythema on face	5	Both resolved	-8
I	45 mg	10	Diagnosis of eczema since 2007 ongoing; red erythema of chest and face	3	Red erythema resolved	-7
K	45 mg	17	Red erythema + dyspigmentation of abdomen, back, hands, scalp	0	Red erythema resolved; some dyspigmentation left	-17
P	90 mg	13	Diagnosis of rash related to SLE since 2016 ongoing; red, scaly erythema on ears, red erythema on nose, shoulder, V-neck area	0	All erythema resolved after 2 doses	-13
V	135 mg	21	Red erythema + scaling + dyspigmentation of arms, chest, ears, hands, nose, neck, shoulders and feet	5	Only dyspigmentation left; red erythema + scaling resolved (besides face, scalp)	-16
W	135 mg	27	Dark red erythema & purple, violaceus crusted hemorrhagic erythema on arms; red erythema + scaling, dyspigmentation & scarring on back, chest, ears, hands, legs, neck, shoulders, scalp; scarring of nose and face	10	Arms only present with red erythema; most erythema have pink instead of red, scaling has decreased	-17

EOT is Day 85 visit. For patient P: Day 85 data were not available, the indicated value is based on the follow-up visit.

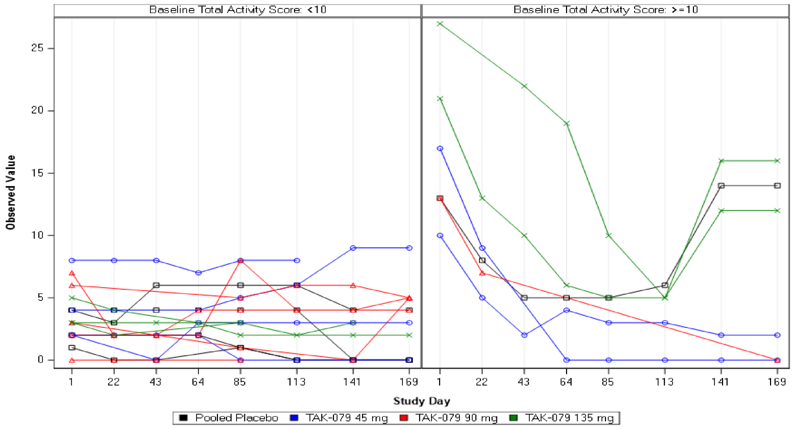

CLASI responders demonstrated notable, clinically meaningful improvements in cutaneous manifestations by the end of treatment. Subgroup analysis for total activity scores revealed that all patients with a baseline CLASI score of >10 met responder criteria at end of treatment.

Supp. Figure 3: Pharmacokinetics of mezagitamab

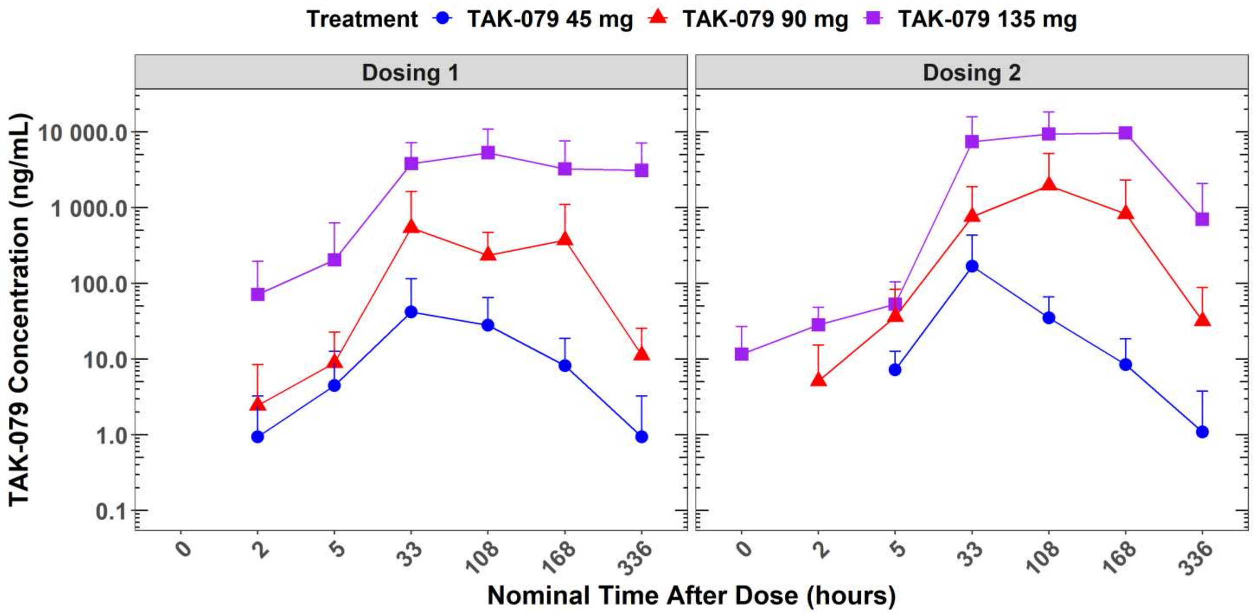

PK of mezagitamab (TAK-079) in SLE patients demonstrated nonlinear increase in exposure in the tested dose range.

Supp. Figure 4: Temporal profiles of IgG, autoantibodies and clinical scores

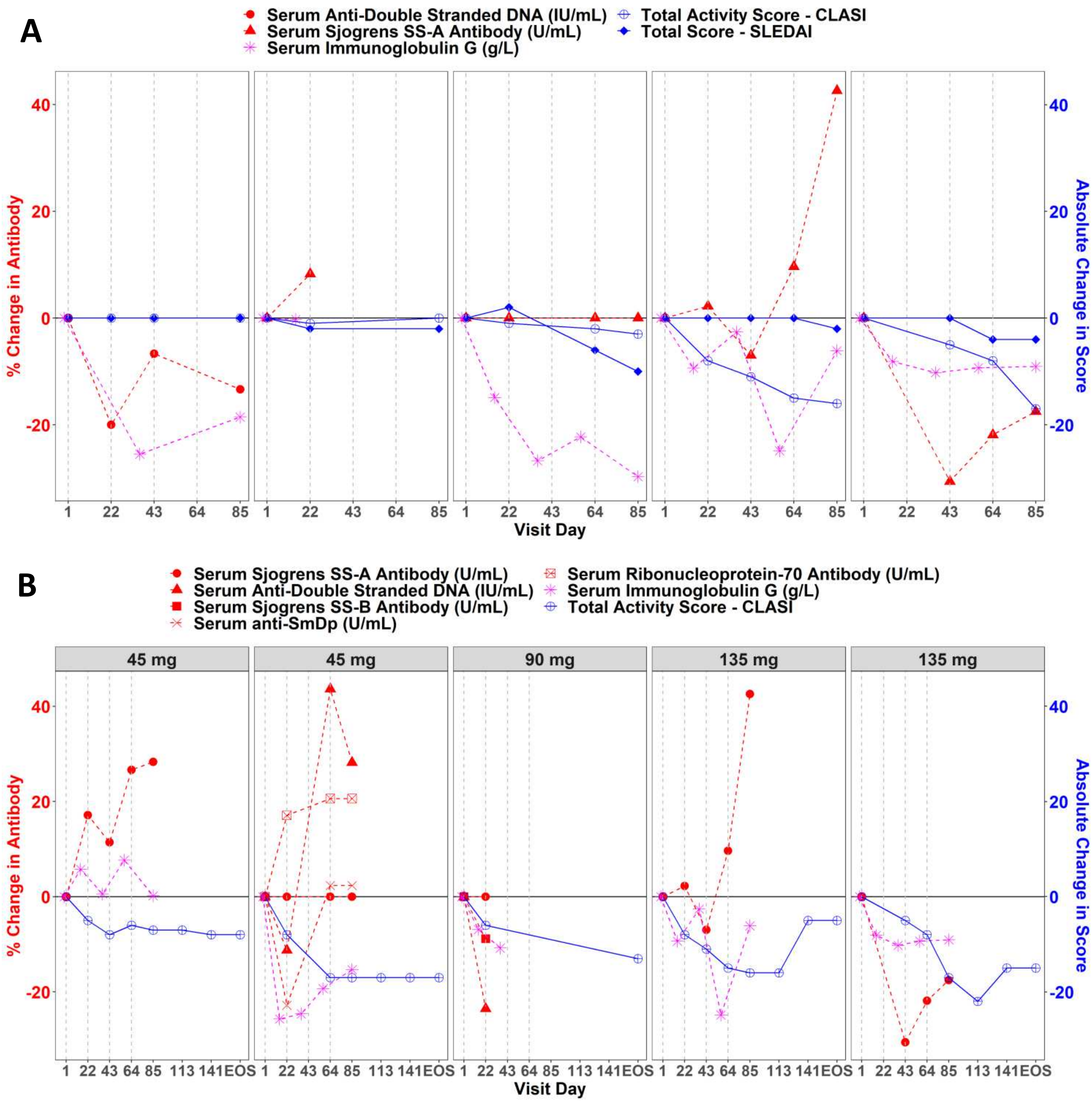

Decreases in serum IgG and autoantibodies were modest and did not appear to be correlated with each other or with clinical response. (A) Data from patients in the highest tested dose of 135 mg, (B) data from CLASI responders in mezagitamab-treated groups.

Supp. Figure 5: 20 cluster CyTOF analysis

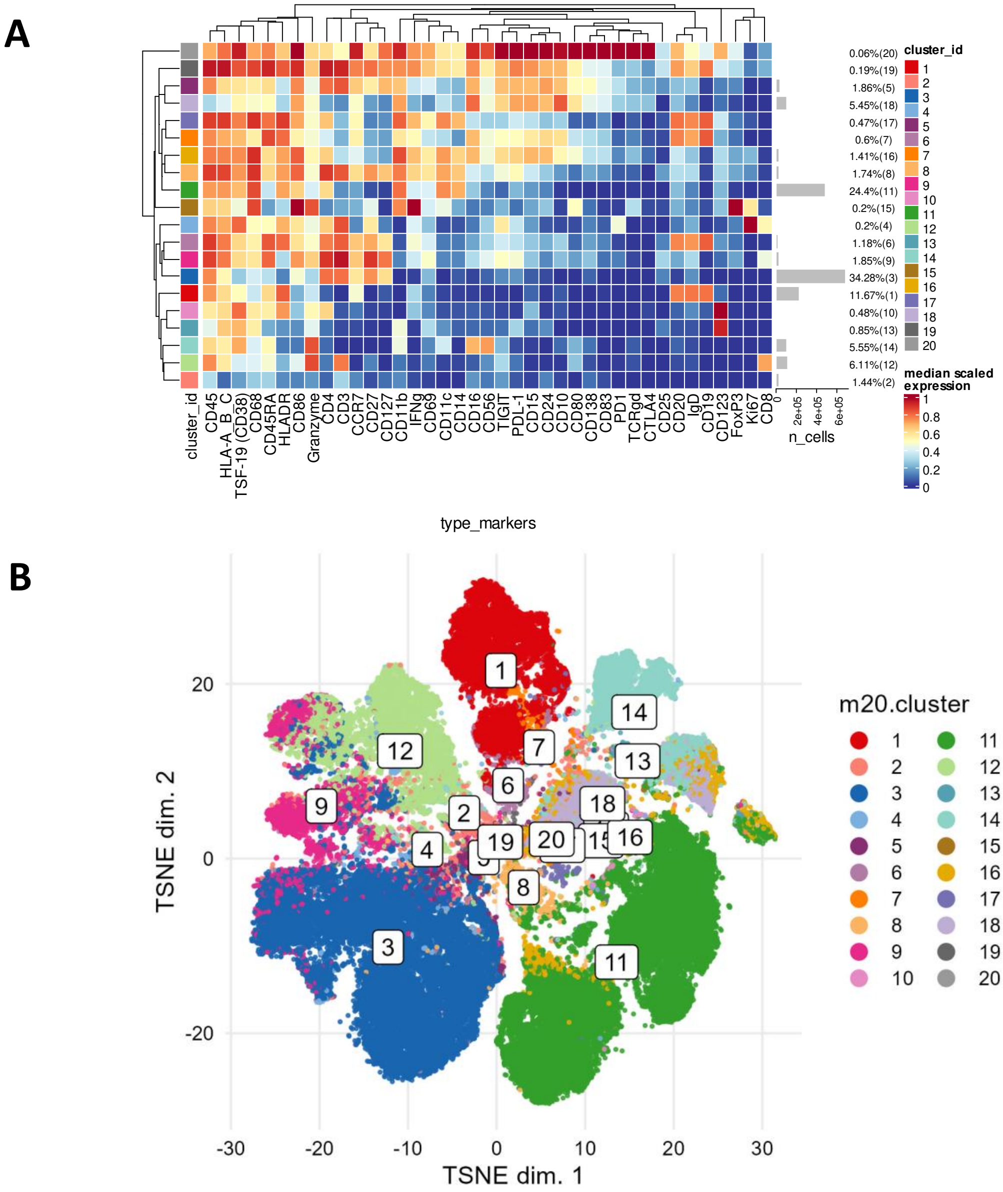

(A) Twenty cluster analysis with phenotypic heatmap of PBMCs from mezagitamab-treated patients, corresponding to clusters identified in (B) labeled TSNE plot.

Supp. Figure 6: 50 cluster CyTOF analysis

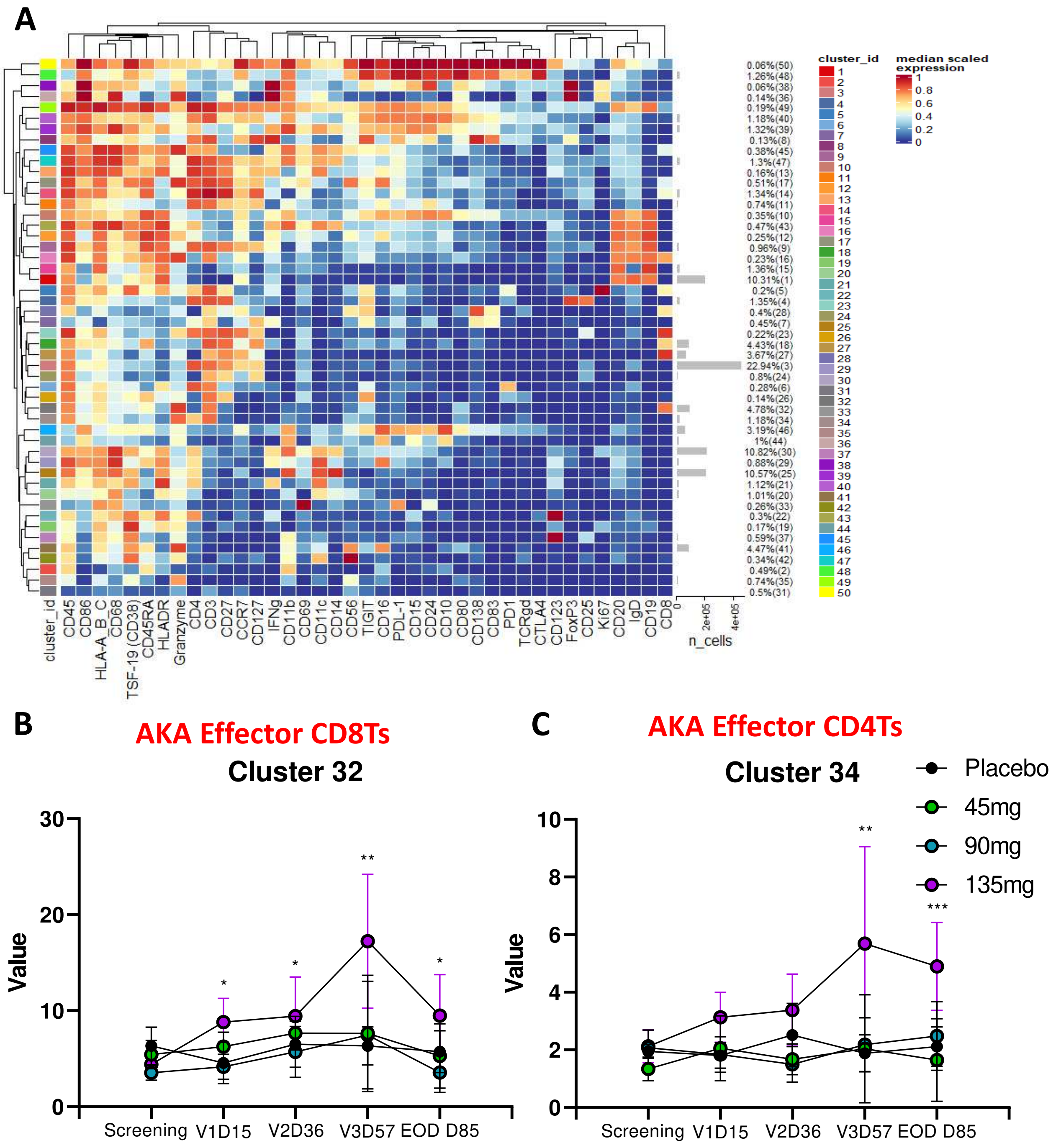

(A) Phenotypic heat map and cluster size (as % of selected events). Treatment effect evaluation identified two clusters with phenotypes consistent with effector-like CD8 T cells (cluster 32, B) and CD4 T cells (cluster 34, C). p-value<0.05 (*), 0.01 (**), or 0.001 (***) of 135 mg dose when compared to placebo.
